# Inhibitory Mechanism of Camellianin A against α-Glucosidase: In Vitro and Molecular Simulation Studies

**DOI:** 10.3390/foods13172835

**Published:** 2024-09-06

**Authors:** Jinze Jia, Lu Bai, Yuzhen Chen, Benguo Liu

**Affiliations:** 1 Henan Institute of Technology, Xinxiang 453003, China; 17630711725@163.com; 2 Digital Agricultural Engineering Research Center of Henan Province, Xinxiang 453003, China; bailu@stu.hist.edu.cn (L.B.); liubenguo@hist.edu.cn (B.L.); 3 Henan Institute of Science and Technology, Xinxiang 453003, China

**Keywords:** camellianin A, α-glucosidase, interaction, fluorescence spectroscopy, molecular docking

## Abstract

α-Glucosidase is an important target for type II diabetes treatment, and the search for natural α-glucosidase inhibitors is currently a hot topic in functional food research. Camellianin A is the main flavonoid in the leaves of *Adinandra nitida*, but research on its inhibition of α-glucosidase is rarely reported. In view of this, the present study systematically investigated the inhibitory impact of camellianin A on α-glucosidase, combining the fluorescence method and molecular docking to explore their interaction, aiming to reveal the relevant inhibitory mechanism. The results indicated that camellianin A possessed excellent α-glucosidase inhibitory activity (IC_50_, 27.57 ± 0.59 μg/mL), and van der Waals force and hydrogen bonding dominated the binding process between camellianin A and α-glucosidase, with a binding-site number of 1. A molecular docking experiment suggested that camellianin A formed hydrogen bonding with Glu771, Trp391, Trp710, Gly566, Asp568, and Phe444 of α-glucosidase, consistent with the thermodynamic result. Our result can provide a reference for the development of natural α-glucosidase inhibitors.

## 1. Introduction

Diabetes is a chronic metabolic disease related to carbohydrate metabolism disorder, and its onset is mainly related to diet and lifestyle. The disease is characterized by symptoms such as high blood glucose, polyuria, polydipsia, and weight loss, severely affecting the health and quality of life of patients [1,2]. Prolonged high blood glucose can also cause protein glycation, leading to complications such as retinopathy, neuropathy, cardiovascular and cerebrovascular diseases, kidney and liver damage, and in severe cases, may result in patient death [3,4,5]. Among the four main types of diabetes, type II diabetes is the predominant type, accounting for over 90% of all cases [6], with the main cause being postprandial hyperglycemia due to insufficient insulin secretion [7,8]. Controlling postprandial blood glucose levels and delaying the progression of long-term diabetes complications are effective treatment methods for achieving normal blood glucose levels. α-Glucosidase is an important metabolic enzyme that releases glucose into the bloodstream by hydrolyzing the glycosidic bonds in carbohydrates, leading to an increase in postprandial blood glucose levels [9]. Therefore, one of the treatment methods for diabetes is to inhibit α-glucosidase to effectively delay the postprandial blood glucose elevation in diabetic patients [10]. Currently, antidiabetic drugs that inhibit α-glucosidase include miglitol, acarbose, and voglibose [11]. However, these microbial and chemically synthesized drugs can cause serious toxic side effects such as hepatotoxicity, diarrhea, and abdominal pain [12,13]. Natural products have always been the most important and efficient source of compounds in drug development, mainly derived from chemical substances in edible or medicinal plants, which are low in cost and relatively safe. Studies have found that various natural compounds, such as flavonoids, polysaccharides, phenolic acids, and terpenes, exhibit good α-glucosidase inhibitory activity [14,15].

**Figure 1:**
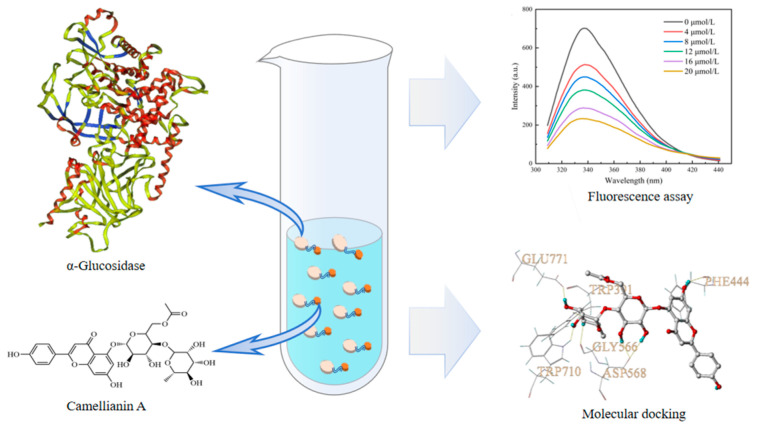
Schematic diagram of the research.

Camellianin A is a natural flavonoid, which is the main flavonoid in a unique healthy tea (Shiyacha) from southern China, with a content exceeding 20% [16]. Camellianin A has many bioactivities such as anti-inflammatory, antioxidant, anticancer, and reducing the risk of cardiovascular diseases [17]. Yuan et al. found that camellianin A possessed good antioxidant activity and could serve as a potential natural antioxidant [18]. Gao et al. found that camellianin A could induce cell apoptosis and inhibit the proliferation of breast cancer MCF-7 cells and liver cancer HepG2 in a dose-dependent manner [19], indicating that camellianin A was a potential lead compound. However, there have been no reports on the inhibitory effect of camellianin A on α-glucosidase. Therefore, this study systematically investigated the inhibitory impact of camellianin A on α-glucosidase, combining fluorescence assay and molecular docking to study their interactions in order to reveal the relevant inhibitory mechanism (Figure 1). The result may provide new insights for the development and application of camellianin A.

## 2. Materials and Methods

### 2.1. Materials and Chemicals

Camellianin A was prepared using our previous method [16]. One hundred grams of *Adinandra nitida* leaves were extracted with 2000 mL of 80% ethanol for 30 min at 70 °C and then filtered. The resulting filtrate was evaporated by vacuum rotation to remove ethanol. The residue was extracted with 2000 mL of boiling water for 30 min and filtered. The filtrate was kept for 24 h at 2 °C and then filtered. The obtained precipitate was subjected to repeated recrystallization through the aforementioned steps of hot dissolution and cold precipitation for a total of ten cycles and freeze-dried to obtain 2.58 g of camellianin A. Acarbose, α-glucosidase (from yeast), and *p*-nitrophenyl-α-D-glucopyranoside (PNP-G) were purchased from Aladdin (Shanghai, China). All other chemicals were of analytical grade.

### 2.2. Determination of α-Glucosidase Inhibitory Effect

According to a previous report [20], the α-glucosidase inhibitory ability was evaluated. α-Glucosidase, camellianin A, and PNP-G were dissolved in PBS buffer solution (0.1 M, pH 6.8) for testing. Then, 1 mL of camellianin A solution at different concentrations (10–100 μg/mL) was mixed evenly with 1 mL of α-glucosidase solution (0.2 U/mL) in a centrifuge tube and incubated at 37 °C for 10 min. PNP-G solution (1 mL, 1 mM) was then added to the tube, and the resulting mixture was incubated at 37 °C for 20 min. Finally, 1 mL of ethanol was quickly added to terminate the reaction, and the absorbance value was measured at 405 nm (A_1_). According to the above steps, 1 mL of PBS was used to replace the sample solution for determining the control absorbance value (A_0_). The α-glucosidase inhibitory activity of the sample (S) was calculated according to Formula (1).

(1) $$S=\frac{A_{0}-A_{1}}{A_{0}}\times 100\%$$

### 2.3. Determination of Binding Constant

According to the report by Li et al. [21], the binding constant between camellianin A and α-glucosidase was measured. Briefly, 4 mL of 2 U/mL α-glucosidase solution was mixed uniformly with 1 mL of camellianin A solution (0–100 µM), and the reaction system was formed by incubating at 30 °C for 30 min. The obtained reaction system was subjected to fluorescence scanning, and the fluorescence emission spectrum (300–450 nm) was collected at an excitation wavelength of 280 nm. The scanning voltage was set at 750 kV, and the widths of the emission and excitation slits were 5 nm. In order to correct the inner filter effect, Formula (2) was used for fluorescence data correction.

(2) $$F=F_{\mathrm{Init}}\times e^{\frac{A_{\mathrm{ex}}+A_{\mathrm{em}}}{2}}$$

F and F_Init_ represent the fluorescence intensity before and after sample correction. A_ex_ and A_em_ are the absorbance of the sample solution at the excitation and emission wavelengths, respectively.

The corresponding quenching rate constant (K_q_) can be calculated based on the following Stern–Volmer Equation (3):

(3) $$\frac{F_{0}}{F}=1+K_{SV}\cdot [Q]=1+K_{q}\cdot \tau_{0}\cdot [Q]$$

where F_0_ and F are the fluorescence intensities before and after the addition of camellianin A; [Q] is the concentration of camellianin A; τ_0_ is the average lifetime of the protein (10^−8^ s).

For static quenching, the binding constant (K_A_) and the binding-site number (n) could be obtained using the double-logarithmic curve Formula (4):

(4) $$\mathrm{lg}[\frac{F_{0}-F}{F}]=\mathrm{lg}K_{A}+n\cdot \mathrm{lg}[Q]$$

### 2.4. Determination of Thermodynamic Parameters

The reaction system of α-glucosidase solution and camellianin A was constructed, and the reaction was carried out at 30 and 37 °C for 30 min in a water bath. Fluorescence spectra were obtained using a fluorescence spectrophotometer to calculate the K_A_ and n values of the reaction system at different temperatures (T). Then, the corresponding entropy change (ΔS) and enthalpy change (ΔH) values could be calculated by fitting the Van’t Hoff Equation (5). Finally, the free energy change (ΔG) at different temperatures could be obtained based on the Gibbs–Helmholtz Equation (6).

(5) $$\ln K_{A}=-\frac{\Delta H}{RT}+\frac{\Delta S}{R}$$

(6) ΔG=ΔH−T⋅ΔS

where R is the gas molar constant (8.314 J mol^−1^ K^−1^)

### 2.5. Measurement of Synchronous and 3D Fluorescence Spectra

According to the method proposed by He et al. [22], the synchronous and three-dimensional fluorescence spectra were recorded. The α-glucosidase solution was mixed with camellianin A solution, and the spectra were scanned at 30 °C. Synchronous fluorescence spectra were recorded at Δλ = 15 nm with an excitation wavelength range of 265–350 nm, as well as at Δλ = 60 nm with an excitation wavelength range of 220–350 nm. Three-dimensional fluorescence spectra were recorded with the excitation wavelength ranging from 220 to 300 nm and the emission wavelength ranging from 300 to 450 nm. The slit widths for excitation and emission were 5 nm.

### 2.6. Molecular Docking

The Surflex-Dock GeomX (SFXC) docking module in SYBYL 8.1 software was applied to construct the binding mode of camellianin A with α-glucosidase, studying their interaction mode and mechanism. The crystal structure of α-glucosidase was from the RCSB PDB database (http://www.rcsb.org/, accessed on 17 January 2024, ID: 4J5T). Water molecules and non-essential substructures were removed, polar hydrogens and charges were added, residues were repaired, and the protein preparation of α-glucosidase was completed. The 3D molecular structure of camellianin A was from the PubChem database (https://pubchem.ncbi.nlm.nih.gov/, accessed on 26 April 2024), and its structure was imported into SYBYL 8.1 software in mol 2 format. The molecule was optimized using the Powell conjugate gradient method, Gasteiger–Huckel charges were loaded, the maximum iteration coefficient was set to 10,000, the energy convergence limit was set to 0.005 kJ/mol, and other parameters were set to default values. Molecular docking was performed using the Surflex-Dock GeomX (SFXC) ultra-high precision docking method. After docking, the conformation with the highest total score and relatively high CScore was selected as the research object [23]. Typically, output conformations with a total score higher than 6 were considered good. The empirical scoring function CScore ranges from 0 to 5, with higher CScore values indicating better selectivity of the output conformation for the molecule.

### 2.7. Statistical Analysis

Each experiment was repeated three times, and the results were presented as the mean ± standard deviation. Graphs were generated using Origin 2018 software.

## 3. Results and Discussion

### 3.1. Inhibitory Effect on α-Glucosidase

**Figure 2:**
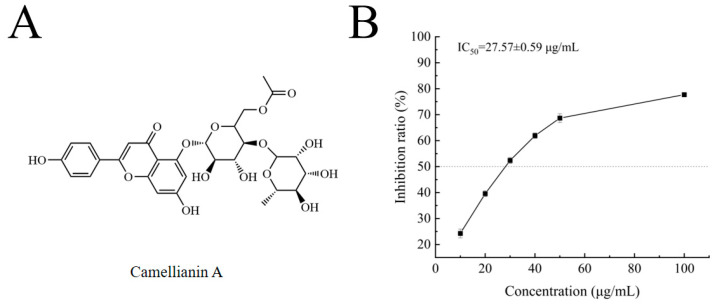
The structure of camellianin A (**A**) and its impact on the activity of α-glucosidase (**B**).

Camellianin A is a natural flavonoid glycoside (Figure 2A) found in the famous folk tea (Shiyacha) from Guangxi Province, China. In previous pharmacological studies, camellianin A has shown strong antioxidant activity and significant anticancer effects on tumor cells, but its α-glucosidase inhibitory ability has not been reported. This study investigated the inhibitory effect of camellianin A on α-glucosidase, with its activity represented by the IC_50_ value [24], which was calculated using Origin 2018 software. As shown in Figure 2B, the inhibition rate of camellianin A on α-glucosidase was significantly positively correlated with concentration, indicating a significant dose-dependent inhibitory effect on enzyme activity. When the concentration is 100 μg/mL, the inhibition rate of camellianin A on the enzyme was 77.71%. The calculated IC_50_ value of camellianin A on α-glucosidase was 27.57 ± 0.59 μg/mL. The inhibitory effect of camellianin A is superior to acarbose (IC_50_ value of >1 mg/mL) [25]. Therefore, Camellianin A is a potential α-glucosidase inhibitor.

### 3.2. Binding Behavior Analysis

The research about the interactions between small molecules and biomacromolecules, especially biologically active small molecules, contributes to understanding the transport and distribution of active ingredients in the body and is of great significance for elucidating their efficacy and mechanisms of action. Fluorescence spectroscopy is an effective method for studying the interactions of protein–ligand complexes. The research mainly includes binding constants, binding-site number, driving force, and conformational changes in protein molecules during the interaction. Aromatic amino acids such as tryptophan, tyrosine, and phenylalanine have strong endogenous fluorescence, making proteins fluorescent [26,27].

**Figure 3:**
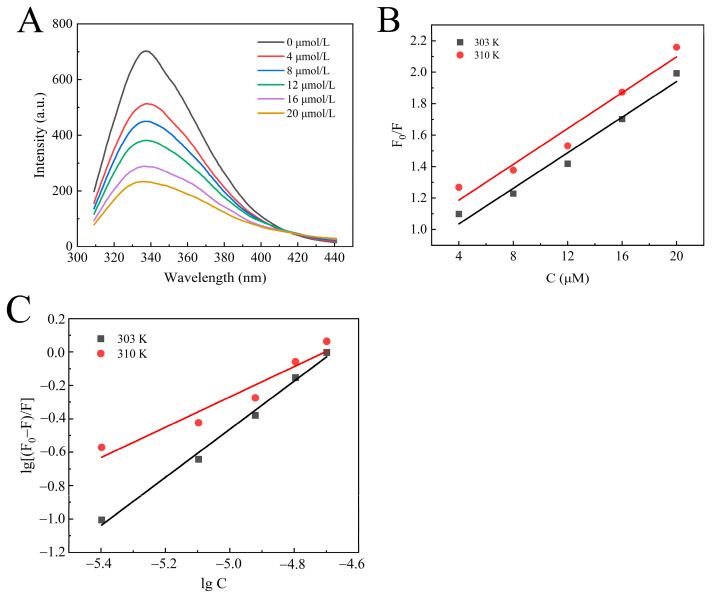
Fluorescence analysis of the interaction between camellianin A and α-glucosidase ((**A**), fluorescence spectra; (**B**), Stern–Volmer graph; (**C**), double-logarithmic graph).

The interaction between enzymes and small molecules can alter the microenvironment of amino acid residues, leading to a decrease in fluorescence intensity. The effect of camellianin A on the fluorescence spectrum of α-glucosidase is shown in Figure 3A. At the excitation wavelength of 280 nm, α-glucosidase exhibited a fluorescence characteristic absorption peak at 338 nm. With increasing concentrations of camellianin A, the fluorescence intensity also gradually declined, with the maximum fluorescence intensity decreasing from 642.644 to 239.602. This indicated that α-glucosidase interacted with camellianin A, quenching the intrinsic fluorescence of α-glucosidase and leading to a decrease in fluorescence intensity. Its characteristic absorption peak shifted from 338 nm to 336 nm, suggesting that the binding of camellianin A with α-glucosidase enhanced the hydrophobicity of the enzyme’s fluorescence groups.

Fluorescence quenching has two different mechanisms: one is dynamic quenching through thermal motion and molecular collision, and the other is static quenching by forming a complex [28]. As shown in Figure 3B, the good linear relationship between F_0_/F and concentration confirms a single quenching process, either static or dynamic quenching [29]. From Table 1, the quenching rate constant (K_q_) values calculated based on the slope were all higher than the maximum collisional quenching rate constant (2.0 × 10^10^ L/mol·s), indicating that the static quenching of α-glucosidase with camellianin A was attributed to the formation of a complex.

In order to further analyze the interaction between α-glucosidase and camellianin A, the corresponding binding constant (K_A_) and the binding-site number (n) values were calculated using double logarithmic curves. Figure 3C demonstrates the double logarithmic curve, and the calculated results are shown in Table 1. Under the conditions of 30 and 37 °C, the K_A_ values of the reaction system were 5.52 × 10^6^ and 1.86 × 10^4^ L/mol·s, respectively. The magnitude of the K_A_ values was greater than 10^4^ Lmol^−1^, confirming a high affinity. The binding-site number at different temperatures was close to 1. As the temperature increases, both the K_A_ and n values of the reaction system decrease. This is because the increase in temperature promotes molecular motion, which is not conducive to the stability of the α-glucosidase/camellianin A complex [30].

Hydrophobic interaction, electrostatic force, hydrogen bond, and van der Waals forces are the primary interactions in protein–ligand complexes. Based on extensive experimental data, the relationship between the thermodynamic parameters of the reaction and the forces involved has been summarized [31,32]. In Table 1, the negative ΔG values at different temperatures indicated that the formation of the α-glucosidase/camellianin A complex was a spontaneous process. Both ΔH and ΔS values of the reaction were negative, which suggested that hydrogen bonds and van der Waals forces dominated the interaction process.

### 3.3. Synchronous and 3D Fluorescence Analysis

**Figure 4:**
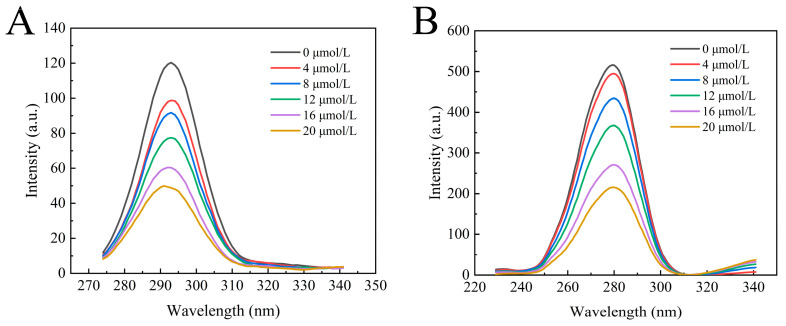
Effect of camellianin A on the synchronous fluorescence spectra of α-glucosidase ((**A**), Δλ = 15 nm; (**B**), Δλ = 60 nm).

Synchronous fluorescence spectroscopy is an effective technique for measuring changes in the protein microenvironment. Fluorescent proteins contain chromophores such as tryptophan, tyrosine, and phenylalanine residues (with a lower quantum yield for phenylalanine residue). When Δλ is 15 nm and 60 nm, characteristic peaks of tyrosine and tryptophan residues can be observed, respectively. Figure 4A,B exhibit the synchronous fluorescence spectra of tyrosine and tryptophan residues in α-glucosidase upon the addition of a series of concentrations of camellianin A. Camellianin A interacted with tryptophan and tyrosine residues of α-glucosidase, resulting in a systematic decrease in synchronous fluorescence. However, the decreasing trend was different, with the decrease in tryptophan fluorescence intensity being greater than that of tyrosine, indicating that the binding site was closer to the tryptophan residue. Additionally, the characteristic peak of the tyrosine residue shifted from 293 nm to 291 nm, suggesting that the complex enhanced the hydrophobicity of the enzyme’s fluorescent groups and reduced polarity.

**Figure 5:**
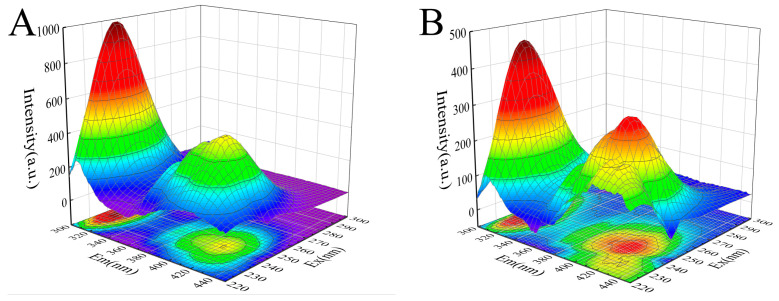
Effect of camellianin A on the synchronous fluorescence spectra of α-glucosidase ((**A**), the fluorescence spectrum of α-glucosidase without camellianin A; (**B**), the fluorescence spectrum of α-glucosidase with camellianin A).

The 3D fluorescence spectrum of proteins can reflect changes in their structure and microenvironment. The intermediate concentration of camellianinA (12 μmol/L) was selected to investigate the effect of camellianinA on the 3D fluorescence spectrum of α-glucosidase. As shown in Figure 5, α-glucosidase exhibited two characteristic peaks (peak 1 and peak 2) in the 3D fluorescence spectrum. Peak 1 (λ = 280 nm) was a typical representative peak of the spectral characteristics involving the π→π* transition of tryptophan and tyrosine residues in enzymes [33], while peak 2 (λ = 226 nm) represented a typical representative peak of the peptide backbone of α-glucosidase [34]. After the addition of camellianin A, the fluorescence intensity of peaks 1 and 2 significantly decreased. Peak 2 exhibited a slight blue shift, indicating that camellianin A affected the microenvironment of the α-glucosidase peptide chain by forming a complex, increasing its hydrophobicity, and leading to changes in the peptide structure.

### 3.4. Molecular Docking Analysis

The interaction between polyphenols and enzymes involves complex structural changes, including higher-order structural rearrangements accompanied by energy transfer and conformational alterations. Currently, various experimental techniques are available for studying these interactions, such as isothermal titration calorimetry, surface plasmon resonance, and nuclear magnetic resonance [35,36,37]. However, the complexity of experimental parameter settings poses significant challenges for research, and most experimental methods struggle to directly obtain dynamic and microscopic information about the interaction process. In recent years, molecular simulation has emerged as an important tool in scientific research. The rapidly advancing ONIOM calculation, molecular dynamics, and molecular docking have been employed to investigate the mechanisms of molecular interactions, yielding substantial results [38,39]. Shukor et al. investigated the interaction between polyphenols and angiotensin-converting enzymes using molecular docking and quantitative structure–activity relationship analysis [40]. Ugarte et al. applied the ONIOM method to clarify the molecular mechanism of glyphosate inhibiting mitochondrial succinate dehydrogenase [41].

**Figure 6:**
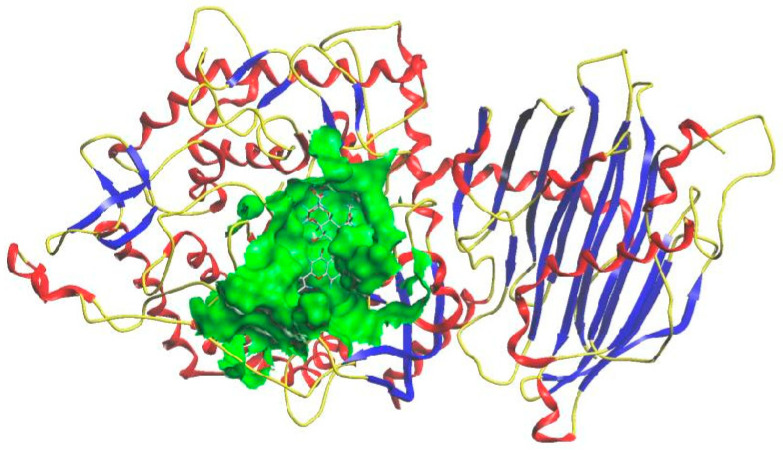
Binding mode of camellianin A and α-glucosidase.

**Figure 7:**
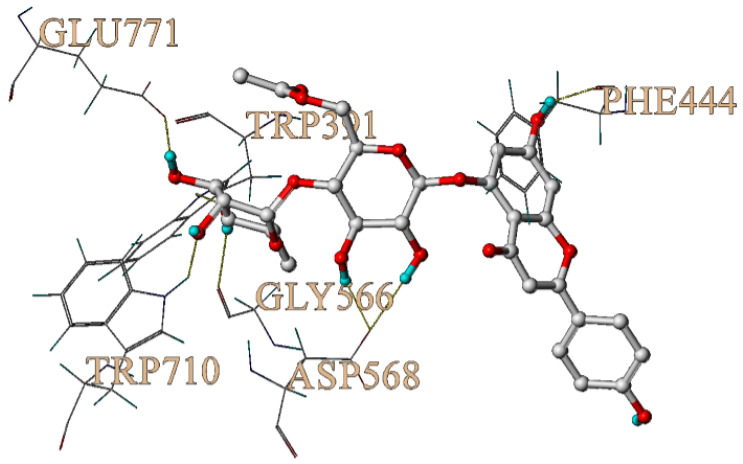
Hydrogen bonding between camellianin A and α-glucosidase.

**Figure 8:**
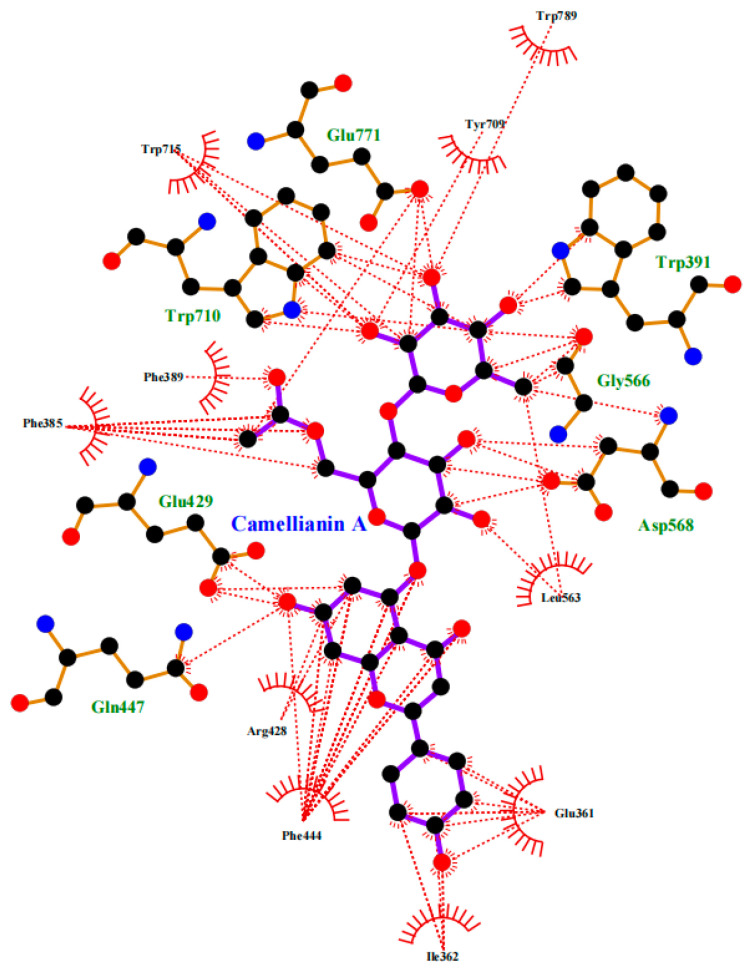
Hydrophobic interaction between camellianin A and α-glucosidase (The dashed line represents the hydrophobic interaction).

In order to further explore the potential interaction mechanism between α-glucosidase and camellianin A, this study utilized the Surflex-Dock GeomX (SFXC) ultra-high precision docking method to explore the binding site of camellianin A. The active site of α-glucosidase was generated in a ligand-like manner, and after molecular docking and scoring, a total of 20 output conformations were obtained. Among all output conformations, the total score scoring was above 8.00, and the conformation with a total score of 9.94 points and a Cscore of 5 was selected (Figure 6). Camellianin A had only one binding site with α-glucosidase, located in the cavity of α-glucosidase. During the binding process, camellianin A formed hydrogen bond interactions with Glu771, Trp391, Trp710, Gly566, Asp568, and Phe444 of α-glucosidase (Figure 7), which coincided with the fluorescence analysis. Camellianin A formed two hydrogen bonds with Asp568 and one hydrogen bond with the remaining residues. In addition, camellianin A also interacted with many surrounding hydrophobic residues (Figure 8). These forces collectively stabilized the interaction between camellianin A and α-glucosidase, effectively competing with substrates for binding sites. The stronger the binding between camellianin A and α-glucosidase, the less binding with substrates, thereby reducing the activity of α-glucosidase.

## 4. Conclusions

This study, for the first time, combined in vitro and molecular simulation methods to reveal the inhibitory mechanism of camellianin A against α-glucosidase. The experimental results indicated that camellianin A had a significant inhibitory effect on α-glucosidase, with an IC_50_ value of 27.57 ± 0.59 μg/mL. Camellianin A could bind to α-glucosidase through van der Waals force and hydrogen bonds, with a binding site number of 1. Molecular docking analysis further confirmed that camellianin A could enter the cavity of α-glucosidase, interacting with Glu771, Trp391, Trp710, Gly566, Asp568, and Phe444 through hydrogen bonds. In addition, it also exhibited hydrophobic interactions with numerous surrounding hydrophobic residues, collectively stabilizing the interaction between camellianin A and α-glucosidase. Our experiments indicate that camellianin A possesses potential α-glucosidase inhibitory activity; however, further investigation of its hypoglycemic effects in in vivo studies is necessary in the future.

## Figures and Tables

**Table 1:** The quenching constant, binding constant, and thermodynamic parameters of the interaction between camellianin A and α-glucosidase.

T (K)	10^12^ K_q_ (L mol^−1^s^−1^)	pK_A_ (Lmol^−1^)	n	ΔG (KJ mol^−1^)	ΔH (KJ mol^−1^)	ΔS (J mol^−1^K^−1^)
303	4.3541	6.7420	1.4409	−39.10	−634.96	−1966.51
303	5.4111	4.2702	0.9079	−25.34		

## Data Availability

The original contributions presented in the study are included in the article, further inquiries can be directed to the corresponding author.
